# Items

**Published:** 1907-04

**Authors:** 


### ITEMS.

---

Messrs. Kress & Owen Company, Manufacturing Chemists, 210 Fulton Street, New York, were visited by fire, on the morning of March 4, 1907, which practically destroyed the manufacturing part of their business. They had, however, a duplicate plant in storage and after four days and nights of continuous work were again turning out *Glyco-Thymoline*. This remarkable display of business enterprise is worthy of record.

---

Messrs. Battle & Co., Saint Louis, have issued recently No. 12 of the series of twelve illustration of intestinal parasites, which they will be pleased to send on application.

